# Editorial: The role of immune cells in tissue regeneration: mechanisms and therapeutic insights

**DOI:** 10.3389/fcell.2025.1730951

**Published:** 2025-11-07

**Authors:** Gaoran Ge, Xianzuo Zhang, Lan Xiao, Jiaxiang Bai

**Affiliations:** 1 Department of Orthopedics, Centre for Leading Medicine and Advanced Technologies of IHM, The First Affiliated Hospital of USTC, Division of Life Sciences and Medicine, University of Science and Technology of China, Hefei, China; 2 Department of Orthopedics, The First Affiliated Hospital of Soochow University, Suzhou, China; 3 School of Medicine and Dentistry, Griffith University, Gold Coast, QLD, Australia

**Keywords:** microenvironment, immune cell, tissue repair, molecular regulation, therapeutic strategies

## Introduction

For a long time, the immune system has been regarded as our “defense force” against pathogens, while tissue regeneration was seen as the exclusive domain of stem cells and progenitor cells. These two fields were once considered entirely distinct. However, the field of regenerative medicine and immunotherapy closely examines the remodeling of the tissue microenvironment, a dynamic process involving changes in cellular interactions, matrix composition, and cytokine levels crucial for tissue repair and regeneration. These insights are fundamentally reshaping our understanding of repair and regeneration. This Research Topic, *The role of immune cells in tissue regeneration: mechanisms and therapeutic insights*, is at the forefront of this revolution. It aims to bring together research from around the globe that eloquently demonstrates the other important roles immune cells play beyond being the first responders after injury.

## Highlights from the research topic

The conventional view regarded inflammation as a harmful process that impedes healing and should be suppressed. However, we now recognize this perspective as one-sided. The immune response, particularly its early inflammatory phase, is a meticulously orchestrated biological program with a defined purpose. When tissue damage occurs, innate immune cells such as neutrophils and macrophages rapidly infiltrate the site. Their role extends far beyond that of mere “scavengers” — clearing dead cells and pathogens. More importantly, by releasing an array of cytokines, chemokines, and growth factors, they construct a unique “immune microenvironment.” This microenvironment acts as a signaling hub, directly instructing resident stem and progenitor cells on whether to remain quiescent, initiate proliferation, or differentiate into functional tissue. Elucidating and further leveraging these mechanisms represents a critical approach to achieving controlled tissue regeneration.

The studies compiled in this Research Topic investigate, discusse, summarize, and analyze the mechanisms of this intricate dialogue across multiple levels. Not only did Pulido et al. elucidate how paracrine signaling guides macrophage polarization toward a pro-regenerative phenotype, but they also directly linked this intercellular dialogue to improved functional outcomes in cardiac remodeling. This establishes a solid theoretical foundation for developing cell-free therapies based on the secretome to treat myocardial infarction. Pu et al. systematically depicted a coordinated transition from pro-inflammatory clearance to anti-inflammatory repair during skeletal muscle injury repair, where stagnation at any stage may lead to regeneration failure. Similarly, Wang et al. dissected the complex cellular network in the liver, an organ with strong regenerative capacity, and elucidated that the activation and fate of hepatic stellate cells (HSCs) are profoundly influenced by a variety of immune cells, including macrophages, which provide multiple potential targets for intervention to reverse liver fibrosis. Li et al. proposed the fundamental idea that the functional state of immune cells is driven by their metabolic programs. Mitochondrial metabolic reprogramming (e.g., the shift from oxidative phosphorylation to glycolysis) is not only a consequence of macrophage polarization, but also the cause of it, which opens up a new dimension of “immune metabolism” for the treatment of chronic inflammation and bone regeneration disorders. With the understanding of the significance of dynamic immunity in the repair of tissue damage, proactive interventions through a wide range of means have become an innovative tool for immunotherapy. Zhang et al. envisions an exciting future in which next-generation biomaterials are intelligent platforms, rather than inert structural scaffolds, that can actively talk to the host immune system. Through their physicochemical properties (e.g., stiffness, topology) and delivery of biologically active molecules, such materials can be designed to recruit pro-regenerative macrophages and other immune cells, thus creating an advantageous “eco-situation” for tissue engineering and regenerative therapies.

This deep mechanistic understanding is giving rise to a new therapeutic paradigm as immune engineering, where the goal is no longer simply to suppress the immune system, but to re-educate and precisely regulate it. We can envision future therapies: designing novel biomaterials that actively recruit and polarize beneficial macrophages; developing nanocarriers that target specific immunomodulatory molecules to the site of injury; and even using single-cell technology to tailor immunomodulatory regimens to maximize a patient’s intrinsic regenerative potential.

## Conclusion

In summary, this Research Topic further expands our understanding of the multiple roles of immune regulation in tissue regeneration. Immune cells are no longer bystanders or disruptors of tissue regeneration but are central players. The effects are felt across a number of key organ systems in the body, including the heart, skeletal muscle, liver and bones. Unraveling the complex molecular dialogue between immune cells and tissue stem cells and taking full advantage of their underlying objective laws is the key to unlocking our own powerful, yet underutilized, regenerative capacity. The road ahead remains challenging and will require close collaboration between immunologists, developmental biologists, clinicians, and bioengineers. There is reason to believe that by harnessing this repair army, we will finally usher in a new era of treatments for the injuries and degenerative diseases that are now considered incurable.

